# Development and Characterization of Potential Ocular Mucoadhesive Nano Lipid Carriers Using Full Factorial Design

**DOI:** 10.3390/pharmaceutics12070682

**Published:** 2020-07-20

**Authors:** Eszter L. Kiss, Szilvia Berkó, Attila Gácsi, Anita Kovács, Gábor Katona, Judit Soós, Erzsébet Csányi, Ilona Gróf, András Harazin, Mária A. Deli, György T. Balogh, Mária Budai-Szűcs

**Affiliations:** 1 Institute of Pharmaceutical Technology and Regulatory Affairs, Faculty of Pharmacy, University of Szeged, Eötvös u. 6, H-6720 Szeged, Hungary; l.kiss.eszter@pharm.u-szeged.hu (E.L.K.); berkosz@pharm.u-szeged.hu (S.B.); gacsi.attila@pharm.u-szeged.hu (A.G.); anita.kovacs@pharm.u-szeged.hu (A.K.); katona@pharm.u-szeged.hu (G.K.); csanyi@pharm.u-szeged.hu (E.C.); 2 Department of Ophthalmology, Faculty of Medicine, University of Szeged, Korányi Fasor 10-11, H-6720 Szeged, Hungary; nyilasne.soos.judit@med.u-szeged.hu; 3 Institute of Biophysics, Biological Research Centre, Temesvári krt. 62, H-6726 Szeged, Hungary; grof.ilona@brc.hu (I.G.); harazin.andras@brc.hu (A.H.); deli.maria@brc.hu (M.A.D.); 4 Doctoral School of Biology, University of Szeged, Dugonics tér 13, H-6720 Szeged, Hungary; 5 Institute of Pharmacodynamics and Biopharmacy, Faculty of Pharmacy, University of Szeged, Eötvös u. 6, H-6720 Szeged, Hungary; balogh.gyorgy@pharm.u-szeged.hu; 6 Department of Chemical and Environmental Process Engineering, Budapest University of Technology and Economics, Műegyetem rakpart 3, 1111 Budapest, Hungary

**Keywords:** nano lipid carrier, mucoadhesion, immunoassay, cell toxicity, cornea PAMPA, penetration studies, Raman mapping

## Abstract

Generally, topically applied eye drops have low bioavailability due to short residence time and low penetration of the drug. The aim of the present study was to incorporate dexamethasone (DXM) into nano lipid carriers (NLC), which contain mucoadhesive polymer, in order to increase the bioavailability of the drug. A 2^3^ factorial experimental design was applied, in which the three factors were the polymer, the DXM, and the emulsifier concentrations. The samples were analyzed for particle size, zeta potential, polydispersity index, and Span value. The significant factors were identified. The biocompatibility of the formulations was evaluated with human corneal toxicity tests and immunoassay analysis. The possible increase in bioavailability was analyzed by means of mucoadhesivity, in vitro drug diffusion, and different penetration tests, such as in vitro cornea PAMPA model, human corneal cell penetration, and ex vivo porcine corneal penetration using Raman mapping. The results indicated that DXM can be incorporated in stable mucoadhesive NLC systems, which are non-toxic and do not have any harmful effect on cell junctions. Mucoadhesive NLCs can create a depot on the surface of the cornea, which can predict improved bioavailability.

## 1. Introduction

Topically applied eye drops are the most commonly used dosage forms in ocular therapy. They are well tolerated by patients but have low bioavailability. The low effective concentration can be explained by the remarkable precorneal loss of drugs, which is due to the elimination mechanisms (tears production and blinking) and ocular barriers. One possibility of overcoming the natural anatomical barrier of the eye is to utilize the surface layer of the mucous membrane as a site of adhesion. This can be done by introducing mucoadhesive agents into the delivery system and increasing the penetration across the mucous layer and membranes. Mucoadhesives can be integrated into various delivery systems, and can be combined with various concepts, such as penetration enhancement and increased solubility. Mucoadhesive drug delivery can be an effective means of delivering ocular drugs as well [1]. Mucoadhesive delivery systems act in two main ways: (i) prolonging residence time at the site of drug absorption and (ii) increasing the epithelial transport of drugs such as peptides and proteins [2].

In the case of topical ophthalmic formulations, a strategy to increase the ocular residence time of active compounds can also be the incorporation of mucoadhesive polymers into the formulations. Mucoadhesives contribute to the formation of a viscous, continuous layer on the surface of the eye that partially protects the drug from elimination by tearing, prolonging the retention time on the eye surface. Different polymers such as carboxymethylcellulose, hydroxypropyl methylcellulose (HPMC), hyaluronic acid or xanthan gum have been used for this purpose in ophthalmic drug deliveries [3,4,5] due to their good tolerance on the eye both in vitro and in vivo [6].

The mucoadhesive approach can be transferred to nanoparticulate systems as well, where the use of mucoadhesive polymers in liposomes and other nanosystem formulations can result in greater efficacy and thus a more successful treatment of ophthalmic diseases [7,8]. This approach can be executed in two main ways: (i) application of a nano-hydrogel hybrid system, where the nanosystem is dispersed in a hydrogel, or (ii) modification of the nanoparticle’s surface with mucoadhesive features (linkage of adhesives and/or modification of the surface charge).

In recent years, the combination of nanocomposites, such as nanoparticles [9], nanoemulsions [10], or niosomes [11], with hydrogels to improve the ophthalmic bioavailability of drugs after topical instillation have been published. In addition, combinations of liposomes and hydrogels are being evaluated by many research teams [7,12]. Among the polymers used to create the mentioned nano-hydrogel hybrid systems, carbopols, chitosans, HPMC, and natural gums have been the most commonly applied. Yu et al., 2015 [12] prepared liposomes filled with timolol maleate and inserted them into a gellan gum gel. This combination increased the duration of action and shortened the onset of action. Lux et al. [13] developed a lyophilized polymer matrix from hydroxypropyl methylcellulose (HPMC), which is located on a flexible poly(tetrafluoroethylene) carrier strip. The bioavailability of fluorescein was measured after loading the lyophilized (or lyophilizator) system into a sachet. Upon contact with the conjunctiva, the lyophilizate was rapidly hydrated by the tear fluid. Compared to conventional eye drops, this new dosage form resulted in significantly higher corneal and aqueous concentrations for 7 h after application. Morsi et al., 2017 [10] prepared an acetazolamide-based nanoemulsion formulation by the inclusion of the combination of several polymers, among them HPMC, in the external phase. The authors observed that HPMC acted as a viscosity enhancer and was able to prolong the intraocular pressure lowering effect in glaucomatous rabbits. This action was attributed to the increased retention time of the nanoemulsion on the ocular surface.

Nanostructured lipid carriers (NLCs), which were functionalized for a mucoadhesive purpose, can be found in the literature with surface modification as well. J. Shen et al. [14] developed a thiolated mucoadhesive polymer coated NLC with cysteine–polyethylene glycol stearate (Cys-PEG-SA) conjugates, administered topically to rabbit eyes. It was found that the encapsulated cyclosporin remains on the ocular surface for up to 6 h, and the precorneal retention time and concentration in the anterior segment increased compared with the NLC without thiomers. 

Liu et al. [15] investigated curcumin containing surface modified NLC systems with thiolated chitosan. They synthesized an N-acetyl-l-cysteine functionalized chitosan copolymer and compared these modified polymer coated NLC systems with the simple chitosan coated NLCs. The modification of chitosan significantly improved transcorneal penetration compared to chitosan hydrochloride coated and noncoated NLCs [15]. Chanburee et al. examined the effect of various polymers on the mucoadhesive properties of different NLC systems. Curcumin-filled NLCs were prepared and the surface of the particles was coated with mucoadhesive polymers: polyethylene glycol 400 (PEG 400), polyvinyl alcohol (PVA), and chitosan (CS). In vitro mucoadhesive assays were evaluated by a wash test, and both PEG-NLC and PVA-NLC showed a strong interaction with porcine mucosa; the mucosal adhesion observed was twice as strong as for uncoated NLCs [16].

Dexamethasone (DXM) is a steroidal anti-inflammatory drug, which is often used after eye surgery to prevent severe injury and inflammatory processes. The water solubility of DXM is low, which is why only suspensions are available in the market. The DXM suspension has very low bioavailability in the anterior segment of the eye because of drainage, lachrymation, poor retention at the absorption surface, and low penetration across the hydrophilic layers [9]. As DXM is a lipophilic drug, its dissolution in an NLC might increase the effectiveness of DXM containing eye drops [17]. Furthermore, NLC formulations might provide better bioavailability with mucoadhesive polymers.

The aim of this study was to incorporate DXM into NLC systems that contain a mucoadhesive polymer (HPMC), where a mucoadhesive gel layer around the nanocarrier can ensure the adhesion of the nanoparticles to the mucosal surface. In our formulation, the mucoadhesive gel layer forms in situ because the amphiphilic characteristics of the polymer can result in its enrichment at the interface of the nanocarrier. A 2^3^ factorial experimental design was made, in which the three factors were the polymer concentration, the DXM concentration, and the emulsifier concentration. The samples were then analyzed for particle size, zeta potential, polydispersity index, and Span value. The significant factors were identified. The biocompatibility of the formulations was evaluated with human corneal toxicity tests and immunoassay analysis. The possible increase in bioavailability was analyzed by means of mucoadhesivity, in vitro drug diffusion, and different penetration tests, such as in vitro cornea parallel artificial membrane permeability assay (PAMPA) model, human corneal cell penetration, and ex vivo porcine corneal penetration using Raman mapping.

## 2. Materials and Methods 

### 2.1. Materials

Compritol 888ATO (glycerol dibehenate) was kindly supplied by Azelis Hungary Ltd. (Budapest, Hungary). Miglyol 812N (capric triglyceride) was provided by Sasol GmbH (Witten, Germany). Kolliphor RH40 (PEG-40 hydrogenated castor oil) was kindly supplied by BASF SE Chemtrade GmbH (Budapest, Hungary). Hydroxypropyl methylcellulose (Methocel F4M) was purchased from Colorcon (Dartford, Kent, England). DXM, DMSO, recombinant human insulin, recombinant human EGF, rat tail collagen, trypsin, ethylenediaminetetraacetic acid solution, collagen type I, Ringer buffer, Ringer-Hepes buffer, fluorescein, Evans blue labeled albumin, 3% paraformaldehyde solution, TX-100 solution, and bovine serum albumin were purchased from Sigma-Aldrich (St. Louis, MO, USA).

### 2.2. Sample Preparation 

For the preparation of the NLC samples, an ultrasonication method was used. The lipids (Compritol 888 ATO and Miglyol 812N at 7:3 ratio) and surfactant (Cremophor RH40) were melted with a heating magnetic stirrer at 85 °C. The total lipid concentration was 10 *w*/*w*%. After that, DXM and the polymer (Methocel F4M) were added to the melted mixture and stirred at a temperature of 85 °C. 

Pure water was heated at 85 °C and the two phases were mixed and ultrasonicated (one cycle, 70% amplitude, 10 min) with a Hielscher UP200S ultrasonic homogenizer (Hielscher Ultrasonics GmbH, Teltow, Germany). The NLC systems were cooled in an ice bath with continuous mixing using a magnetic stirrer. Table 1 includes the compositions of the NLCs prepared.

### 2.3. X-ray Diffraction (XRD) Analysis of the Lyophilized NLCs

The solid state of DXM in the lyophilized lipid composition was analyzed with XRD. After the sample preparation, the formulations were freeze-dried.

In the case of NLC systems, freeze-drying was performed in Scanvac CoolSafe 100-9 Pro type equipment (LaboGene ApS, Lynge, Denmark). The liquid samples were transferred to 1.5 mL vials and freeze-dried at −40 °C for 24 h under 0.013 mbar pressure and then kept at 25 °C for 12 h as secondary drying to obtain lyophilized powders. The process was controlled by a computer program (Scanlaf CTS16a02); the temperature of the product and pressure values were monitored continuously.

The XRD measurements were performed with a Bruker D8 Advance diffractometer (Bruker AXS GmbH, Karlsruhe, Germany). The Cu K λI radiation (λ = 1.5406 Å) was used. The samples were scanned from 3° to 40° 2θ at 40 kV and 40 mA. The scanning speed was 0.1°/s, the step size was 0.010°.

### 2.4. Particle Size and Zeta Potential

The hydrodynamic diameter (Z_ave_), zeta potential (ZP), and polydispersity index (PI) of NLCs 1-8 were investigated by the Zetasizer Nano ZS instrument (Malvern Instruments, Worcestershire, UK) with DTS 1070 folded capillary cell at 25 °C. The applied medium was purified water. The refractive index (RI) was 1.3553. Three parallel measurements were made and 6-fold dilution was used.

The NLCs (Table 1) were also analyzed by particle size and particle size distribution measurements using laser diffraction, Mastersizer 2000 Hydro tool (Malvern Instruments, Worcestershire, UK). The d(0.1), d(0.5), and d(0.9) were assessed. The measuring medium was purified water with RI of 1.33. The RI of the samples was 1.36 and the imaginary RI was 0.01. The Span value can be calculated with the following Equation (1):

(1) $$Span=\frac{d\left(0.9\right)-d\left(0.1\right)}{d\left(0.5\right)}$$

In order to evaluate the stability of NLCs, the particle size and ZP measurements were repeated after 1 week.

### 2.5. Entrapment Efficacy

The entrapment efficacy (EE%) of the NLCs was determined with this method. The clear aqueous phase of the NLCs was separated by centrifugation in Vivaspin 15R 5000 MWCO Hydrosart tubes (Sartorius, Stonehouse, UK) with Hermle Z323K, (HERMLE Labortechnik GmbH, Wehingen, Germany). Centrifugation was performed at 5000 rpm (9000 rcf) for 30 min at 4 °C. 

The DXM content of the filtered solution was examined by HPLC (Shimadzu Nexera X2 UHPLC, Kyoto, Japan), which was equipped with a C18 reverse-phase column (Phenomenex Kinetex C18, Phenomenex, Torrance, CA, USA) with dimensions of 2.6 µm, 100 Å, 150 × 4.6 mm. The separation was evaluated with gradient elution with the following program: time (min)/% of acetonitrile: 0/35, 4.0/60, 4.01/35, 6.0/35, and the detection was made at 240 nm. The injection volume was 5 µL and the flow rate was 1 mL/min. The column temperature was 40 °C. The time of analysis was 6 min and the retention time for DXM was 3.6 min.

The following equation was used to calculate *EE*% (2):

(2) $$EE\;\%=\frac{W_{initial\;drug}-W_{free\;drug}}{W_{initial\;drug}}\times 100$$

### 2.6. Mucoadhesion Study

The mucoadhesion of the formulations (Table 1) and the polymer-free version of the compositions was investigated with a TA.XT plus Texture Analyzer (Stable Micro Systems Ltd., Vienna Court, Lammas Road, Godalming, Surrey, UK. GU7 1YL). A cylinder probe with a diameter of 1 cm [18,19] and a 1 kg load cell were used. Samples were placed in contact with a filter paper disc wetted with 50 µL of an 8% *w*/*w* mucin dispersion as in vitro mucosal surface [19]. The mucin dispersion was made with PBS buffer (pH = 7.4). Five parallel measurements were made. Twenty mg of the sample was attached to the fixed filter paper of the cylinder probe and placed in contact with the artificial mucosal surface. A 2500 mN preload was applied for 3 min. The cylinder probe was moved upwards to separate the sample from the substrate at a prefixed speed of 2.5 mm min^−1^. The work of adhesion (A, mN mm) was used to characterize the mucoadhesive behavior, which was calculated as the area (AUC) under the ‘‘force vs. distance’’ curve. 

### 2.7. In Vitro Drug Release Study

To investigate in vitro drug release, the dialysis bag method was used. Firstly, 200 µL of the samples (NLC1-8) were filled in a Spectra/Por^®^ 4 dialysis membrane (Spectrum Laboratories, Inc., Rancho Dominguez, CA, USA), with Spectra/Por^®^ Closures (Spectrum Laboratories, Inc.). From each composition of NLCs, 3 replicates were used and placed into 20 mL of phosphate-buffered saline (PBS) (PBS of pH = 7.4 was prepared by dissolving 8 g dm^−3^ NaCl, 0.2 g dm^−3^ KCl, 1.44 g dm^−3^ Na_2_HPO_4_·2H_2_O and 0.12 g dm^−3^ KH_2_PO_4_ in distilled water, the pH was adjusted with 0.1 M HCl). The sink condition was ensured during the experiments. The systems were held at 35 °C to mimic in vivo conditions and stirred at 370 rpm with a magnetic stirrer. During the diffusion study, seven sampling times were used (0.5, 1, 2, 3, 4, 5, 6 h) by removing 1 mL from the acceptor phase and replacing it with fresh thermostated PBS. As reference preparation, a DXM suspension (0.1 and 0.05 *w*/*w*%) was formulated (d(0.5) = 7.8 µm measured by Mastersizer 2000) similarly to DXM suspensions in the market. The diffused amounts of DXM were analyzed by HPLC (the HPLC method is described in Section 2.10).

### 2.8. Penetration Study on Corneal-PAMPA Model

For the in vitro transcorneal permeability measurement, the previously reported corneal-PAMPA method was applied [20]. DXM and its formulations were used as a donor solution. In order to create the lipid membrane, phosphatidylcoline (PC, 16 mg) was dissolved in a solvent mixture (70% (*v*/*v*) hexane, 25% (*v*/*v*) dodecane, 5% (*v*/*v*) chloroform), and then each well of the donor plate (MultiscreenTM-IP, MAIPN4510, pore size 0.45 mm; Millipore) was coated with the lipid solution (5 μL each). Then hexane and chloroform were evaporated to form a PC lipid membrane with the concentration of 10.67 *w*/*v*% in each well. The donor plate was then fit into the acceptor plate (Multiscreen Acceptor Plate, MSSACCEPTOR; Millipore) containing 300 μL of PBS solution (pH 7.4), and 150–150 μL of the PBS solutions were put on the membrane of the donor plate. The donor plate was covered with a sheet of wet tissue paper and a plate lid to avoid evaporation. The plates were incubated for 4 h at 35 °C (Heidolph Titramax 1000) followed by the separation of PAMPA sandwich plates and the determination of the concentrations of the APIs in the donor and acceptor solutions by HPLC method (Section 2.5). Test solutions from PAMPA experiments were prepared in 96-well plates and sealed before injection. For each assay, 3 replicates per compound were measured. The effective permeability and membrane retention of drugs were calculated using Equation (3) [20]:

(3) $$P_{e}=-\frac{2.303\;\cdot V_{A}}{A\left(t-\tau_{SS}\right)}\cdot \log\left[1-\frac{c_{A}\left(t\right)}{S}\right]$$

where *P_e_* is the effective permeability coefficient (cm s^−1^), *A* is the filter area (0.24 cm^2^), *V_A_* is the volume of the acceptor phase (0.3 cm^3^), *t* is the incubation time (s), *τ_SS_* is the time to reach steady-state (s), *C_A_*(*t*) is the concentration of the compound in the acceptor phase at time point t (mol cm^−3^), S is the free drug content of DXM in the donor phase.

### 2.9. Human Corneal Epithelial Cell (HCE-T) Line

Human corneal epithelial cells (HCE-T; RCB 2280; RIKEN BRC, Tsukuba, Japan) were immortalised by transfection with a recombinant SV40-adenovirus vector, established and characterized by Araki-Sasaki [21]. The cells were grown in Dulbecco’s Modified Eagle’s Medium/F-12 (Gibco, Life Technologies, Carlsbad, CA, USA) supplemented with 10% fetal bovine serum (Gibco, Life Technologies, Carlsbad, CA, USA), 0.5% DMSO, 5 µg/mL recombinant human insulin and 10 ng/mL recombinant human EGF in a humidified incubator with 5% CO_2_ at 37 °C. All plastic surfaces were coated with 0.05% rat tail collagen in sterile distilled water before cell seeding in culture dishes. The culture medium was changed every second day. When cells reached approximately 80–90% confluence in the dish, they were subcultured with 0.05% trypsin–ethylenediaminetetraacetic acid solution.

### 2.10. Cell Viability Measurements

Real-time cell electronic sensing was used to follow cell damage and/or protection in living barrier forming cells. The RTCA-SP instrument (ACEA Biosciences, San Diego, CA, USA) registered the impedance of cell layers every 10 min and at each time point the cell index was defined as (Rn − Rb)/15, where Rn is the cell-electrode impedance of the well when it contains cells and Rb is the background impedance of the well with the medium alone. The 96-well E-plates with built-in gold electrodes were coated with collagen type I (50 μg/mL for both) and dried for 20 min under sterile air-flow. Culture medium (60 μL) was added to each well for background readings, then 50 µL of HCE-T suspension was dispensed at the density of 5 × 103 cells/well. When cells reached a steady growth phase, they were treated.

For cell viability measurements, the originally prepared liquid formulations such as NLC1, NLC2, NLC3, and NLC4 (Table 1) formulations and HPMC solution were prepared as 10×, 30× and 100× dilutions in cell culture media.

### 2.11. Immunohistochemistry

Morphological changes in HCE-T cells were investigated by immunostaining for junctional proteins zonula occludens protein-1 (ZO-1), occludin, β-catenin and E-cadherin. Cells were grown on culture inserts used for permeability experiments. After the treatment, the inserts were washed with PBS and the cells were fixed with 3% paraformaldehyde solution for 15 min at room temperature. The cells were permeabilized by 0.2% TX-100 solution for 10 min and the nonspecific binding sites were blocked with 3% bovine serum albumin in PBS. Primary antibodies rabbit anti-ZO-1 (AB_138452, 1:400; Life Technologies, Carlsbad, CA, USA), rabbit anti-β-catenin (AB_476831, 1:400), rabbit anti-occludin (AB_2533977, 1:100; Life Technologies, Carlsbad, CA, USA) and mouse anti-E-cadherin (AB_397580, 1:400; Life Technologies, Carlsbad, CA, USA) were applied as overnight treatment. Incubation with secondary antibodies Alexa Fluor-488-labeled anti-mouse (AB_2534088, 1:400; Life Technologies, Invitrogen, Carlsbad, CA, USA) and anti-rabbit IgG Cy3 conjugated (AB_258792, 1:400) lasted for 1 h. Hoechst dye 33342 was used to stain cell nuclei. After mounting the samples (Fluoromount-G; Southern Biotech, Birmingham, AL, USA), staining was visualized by a confocal laser scanning microscope (Olympus Fluoview FV1000, Olympus Life Science Europa GmbH, Hamburg, Germany).

### 2.12. Permeability Study on Cell Culture Model

Transepithelial electrical resistance (TEER) reflects the tightness of the intercellular junctions closing the paracellular cleft, therefore the overall tightness of cell layers of biological barriers. TEER was measured to check the barrier integrity by an EVOM volt-ohmmeter (World Precision Instruments, Sarasota, FL, USA) combined with STX-2 electrodes, and was expressed relative to the surface area of the monolayers as Ω × cm^2^. TEER of cell-free inserts was subtracted from the measured data. Cells were treated when the cell layer had reached steady TEER values.

HCE-T cells were seeded at a density of 10^5^ cells onto Transwell inserts (polycarbonate membrane, 0.4 µm pore size, 1.12 cm^2^ surface area; 3401, Corning Life Sciences, Tewksbury, MA, USA) and cultured for 5–8 days at liquid-liquid and for 4–6 days at air-liquid interface. The culture medium was changed and TEER was checked every second day.

For the permeability experiments, the inserts were transferred to 12-well plates containing 1.5 mL Ringer buffer in the acceptor (lower/basal) compartments. In the donor (upper/apical) compartments, 0.5 mL buffer was pipetted containing different formulations of DXM (10-times dilution of NLCs). To avoid unstirred water layer effect, the plates were kept on a horizontal shaker (120 rpm) during the assay. The assay lasted for 60 min. At a 30-min timepoint, the inserts were transferred to a new well containing Ringer-Hepes buffer. Samples from both compartments were collected and the DXM concentration was detected by HPLC.

To determine the tightness of the cornea epithelial culture model, two passive permeability marker molecules were tested [22]. In the donor compartments 0.5 mL buffer containing fluorescein (10 μg/mL; Mw: 376 Da) and Evans blue labeled albumin (167.5 μg/mL Evans blue dye and 10 mg/mL bovine serum albumin; MW: 67.5 kDa) was added. The inserts were kept in 12-well plates on a horizontal shaker (120 rpm) for 30 min. The concentrations of the marker molecules in the samples from the compartments were determined by a fluorescence multiwell plate reader (Fluostar Optima, BMG Labtechnologies, Germany; fluorescein: excitation wavelength: 485 nm, emission wavelength: 520 nm; Evans blue labeled albumin: excitation wavelength: 584 nm, emission wavelength: 680 nm). 

The apparent permeability coefficients (P_app_) were calculated as described previously [22]. Briefly, cleared volume was calculated from the concentration difference of the tracer in the acceptor compartment (Δ[*C*]*_A_*) after 30 min and donor compartments at 0 h ([*C*]*_D_*), the volume of the acceptor compartment (*V_A_;* 1.5 mL), and the surface area available for permeability (*A*; 1.1 cm^2^) using Equation (4):

(4) $$P_{\mathrm{app}}\;(\mathrm{cm}/s)\;=\frac{\Delta {\left[C\right]}_{A}\;\times \;V_{A}}{A\times {\left[C\right]}_{D}\times \Delta t}$$

For permeability measurements, NLC5, NLC6, NLC7, and NLC8 formulations were applied at 10 times dilution and DXM at 1 mg/mL concentration, all diluted in Ringer-Hepes buffer.

### 2.13. Experimental Design

In order to characterize the polymer containing NLC compositions, a 2^3^ full factorial design was applied, which is suitable for generating a first-order polynomial model (Equation (5)) and for the investigation of the linear response surface. The model describes the principal effects and interaction among the identified variables.

(5) $$y=a_{0}+a_{1}x_{1}+a_{2}x_{2}+a_{3}x_{3}+a_{12}x_{1}x_{2}+a_{23}x_{2}x_{3}+a_{13}x_{1}x_{3}$$

where *a*_0_ is the intercept, *a*_1,2,3_ were the regression coefficients values. *x*_1_*, x*_2_, and *x*_3_ correspond to factors A, B and C, respectively.

The independent factors were A (polymer concentration), B (DXM concentration), and C (surfactant concentration). The optimization parameters were indicated in each measurement, when they were applicable. The chosen factors were examined at two levels (+1 and −1), which corresponds to the values in Table 1.

### 2.14. Penetration Study on Porcine Cornea

The ex vivo penetration test was examined with Raman microscopy. The pig conjunctiva came from a slaughterhouse and was kept at −20 °C until measurement. The porcine cornea was placed on a sterile cotton wool bed moistened with physiological saline solution and the cornea was surrounded by a Teflon ring to prevent the flow of eye drops into the conjunctiva. The cornea was instilled with 250 µL of NLC sample (NLC6 and the polymer-free version of NLC6) every 30 min and the formulation was removed just before the next instillation. The duration of treatment was six hours. The system was thermostated at 35 °C. The treated cornea was frozen and divided into cross sections (20 μm thick) onto aluminum-coated slides using a Leica CM1950 cryostat (Leica Biosystems GmbH, Wetzlar, Germany). Untreated porcine cornea was used as a reference. Microscopic measurements of Raman were performed with a Thermo Scientific DXR Raman microscope (Thermo Fisher Scientific, Waltham, MA, USA). We used 780 nm laser light with a maximum power of 24 mW. The microscopic lenses used for the measurement were magnified 50× and the aperture was 25 mm. The chemical mapping area examined was 150 × 1200 μm; the size of the step was 50 μm vertically and horizontally. A total of 72 spectra were recorded, 16 images were collected for each spectrum, and the exposure time was 5 s. The operation of the instrument and evaluation of the measurements were performed by OMNIC for the Dispersive Raman 8.2 software package (Thermo Fisher Scientific). The characteristic peaks of the DXM spectrum coincided with the characteristic peaks of the corneal tissue; however, the profiled map showed a detectable NLC6 signal. Therefore, during the evaluation, profiling of the Raman map was performed using the entire NLC spectrum.

### 2.15. Statistical Analysis

All data presented are means ± SD. For the factorial design and mucoadhesive studies, statistical data analysis was performed using Statistica for Windows, version 10. In the case of cell penetration tests, the values were compared using one-way ANOVA followed by Dunnett’s test, 2-way ANOVA followed by the Bonferroni test with GraphPad Prism 5.0 software (GraphPad Software, Inc., San Diego, CA, USA). A level of *p* ≤ 0.05 was taken as significant, *p* ≤ 0.01 as very significant, and *p* ≤ 0.001 as highly significant.

## 3. Results and Discussion

### 3.1. Characterization of DXM-Loaded NLCs with Factorial Experimental Design

Our aim was to optimize a DXM containing mucoadhesive NLC system, which is stable, has a narrow size distribution, good entrapment efficacy and enhanced mucoadhesivity. Most of the excipients were chosen according to our previous study [17]; the solid lipid was Compritol 888 ATO, the oil component was Miglyol 812N, and the surfactant was Cremophor RH40. HPMC was applied as a mucoadhesive agent, which supposedly forms a gel layer around the nanoparticles. A 2^3^ full factorial experimental design was used to optimize the formulations, which is able to generate a first-order polynomial model. The model can describe the principal effects and interaction among the identified variables. The chosen factors were polymer concentration (A), surfactant concentration (B), and DXM concentration (C). The optimization parameters (dependent factors) for the characterization of nanoparticles, the particle size (Z_ave_), zeta potential (ZP), polydispersity index (PDI), d(0.1), d(0.5), d(0.9), Span value, entrapment efficacy (EE%), and mucoadhesivity of the NLC systems were chosen. 

#### 3.1.1. XRD Analysis of Lyophilized NLCs

To investigate the solid state of the API in the lipid matrix, XRD measurements were applied. The presence of the characteristics peaks of the API can indicate its crystalline state, while their absence can suggest its amorphous or molecularly dispersed state in the lipid matrix. 

**Figure 1:**
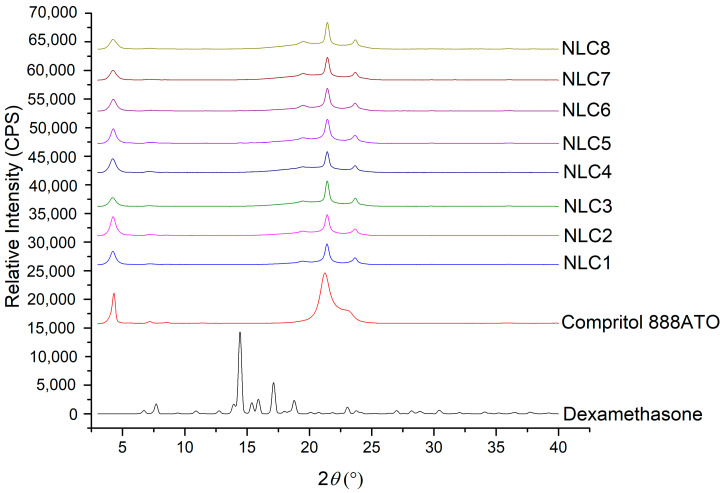
XRD diffractogram of the NLC1-8 compositions, Compritol 888 ATO and DXM.

The diffractograms of DXM powder and Compritol 888 ATO were investigated and compared with the diffractogram of lyophilized NLCs (Figure 1). The X-ray diffraction pattern of DXM showed major peaks at 2θ = 6.1, 9.1, 10.9, 12.86, 14.66, 15.4, and 17.02. None of the characteristic peaks of DXM could be detected in the case of NLC systems, which can indicate the amorphous or molecularly dispersed form of the drug in the nanoparticles.

On the other hand, the characteristic peaks of the solid lipids (the characteristic peaks of Compritol 888 ATO are 2θ = 4.27, 21.25, 22.97) can be found in the lipid mixtures, indicating the presence of some crystalline solid lipid in all cases, which may contribute to increasing entrapment efficacy.

#### 3.1.2. Characterization of NLCs by Zetasizer and Laser Diffraction Method

The emulsifier is essential for the formulation of NLC systems, but it can be one of the main irritating and toxic components of the formulations. For this reason, an effort should be made to minimize the amount of surfactant. In the factorial design, we investigated the two levels of emulsifier (5% and 2.5%) and DXM (0.05% and 0.1%) concentrations; the latter ones correspond to the amount in the eye drops available on the market. The polymer concentration is also essential because too high polymer concentrations can result in a gel form, which makes it impossible to use the formulation as an eye drop. However, the polymer is required to provide the lipid particles with a mucoadhesive effect. In our case, the polymer has amphiphilic behavior [23]; therefore, it can be present at the lipid-water interface in a higher concentration, thus resulting in a concentrated gel layer around the lipid particles. The polymer as a surfactant can modify the accumulation of other surfactants (e.g., Cremophors) at the interface, which may have an effect on the formation and stability of NLCs. The polymer concentration was also investigated at two levels (0.05% and 0.1%).

Z_ave_ = 153.22 − **4.93A** − **42.65B** − **1.74C** − **5.50AB** + **5.71AC** − 0.35BC, (6)

PDI = 0.30 − 0.03A − 0.04B − 0.02C − 0.01AB + 0.04AC + 0.00BC,(7)

ZP = −7.65 + 0.46A + 2.21B − 0.44C + 0.24AB + 0.83AC − 1.49BC,(8)

EE% = 88.63 − 0.51A + 3.6B + 0.35C − 0.69AB + 0.51AC − 1.18BC,(9)

d(0.5) = 0.12 − 0.001A − 0.003B + 0.002C + 0.000AB + 0.000AC + 0.002BC,(10)

Span value = 1.24 − 0.05A − 0.1B + 0.11C − 0.09AB + 0.01AC + 0.1BC.(11)

Equations (6)–(11) show the effect of the chosen factors on the optimization parameters and the combined factors on optimization parameters (Zave, PDI, ZP, EE%, d(0.5) and Span value). The bold values in the equations indicate a significant effect.

The particle size and particle size distribution of the NLCs were investigated with two different techniques. The first focuses on the nano range and can measure the hydrodynamic diameter of the particles, including the possible gel layer around the particles (Table 2). The second method can detect a broader size range, where micro-size aggregates of the nano lipids can also be present (Table 3). The gel layer around the particles may promote aggregation; therefore, we devoted particular attention to following the formation of aggregates.

ZP can be an indicator of the stability of colloid dispersions. The magnitude of ZP indicates the degree of electrostatic repulsion between adjacent, similarly charged particles in the dispersion. The high absolute value of ZP can provide good stability for nanoparticles, while these dispersions have moderate aggregation. If the absolute value of ZP is low, the attractive forces may exceed this barrier and the dispersion may break and flocculate. 

EE% provides information about the amount of DXM which can be incorporated into the lipid matrix of the NLC. The amount of free and entrapped drug can be crucial for the effectiveness of the formulation as a hydrophilic eye drop. Free API can cause a burst effect, while the entrapped API can be attached to the mucosal surface. The slow diffusion from the lipid particles on the mucosal surface can result in prolonged drug release. 

Based on the results of the factorial design, surfactant concentration, polymer concentration and DXM concentration have a significant effect on Z_ave_ (Equation (6)). The combined effects of the polymer and surfactant concentration (AC), and the polymer and DXM concentration (AB) also have a significant effect on Z_ave_. Based on the coefficient values, the surfactant concentration, polymer concentrations, and DXM concentrations are inversely related to particle size (Equations (6) and (10)), which means that the increase in the concentration of the above-mentioned components decreases the particle size of the nanocarriers.

After one week, the particle size of all compositions increased. The slight increase of Z_ave_ can be explained by the formation of a gel layer around the particle, while the remarkable change in the d(0.5), d(0.9) and Span values can indicate the aggregation of the lipid particles via the gel surfaces. In the case of NLC4 and NLC8, the increase in the d(0.5), d(0.9), and Span values was not remarkably higher compared with the other formulations, which can mean that the particles of NLC4 and NLC8 do not aggregate so much even after one week. On the other hand, the Z_ave_ value of NLC4,8 increased to the same extent as for the other formulations, suggesting the formation of a gel layer around the nanoparticles in each composition. NLC4,8 contain a higher amount of surfactant and polymer, and their combination can result in a more stable interfacial layer.

The compositions with a higher surfactant concentration (NLC3,4 and NLC7,8) have a lower absolute ZP value. However, after one week, if the surfactant concentration is higher, ZP will be higher (of absolute value) as well. The reason for this might be that the formation of the final interface structure takes more time than one day. 

The formulations with a lower surfactant concentration (NLC1,2 and NLC5,6) have lower entrapment efficacy. This finding suggests that a higher surfactant concentration is needed to dissolve and entrap DXM in the lipid carriers; 5% of surfactant can result in high, more than 90% EE%.

The absolute ZP value of the nanoparticulated system is quite low, but on the basis of our results the preparation with the lowest absolute ZP values showed the highest stability (considering the Z_ave_, PDI, d(0.5) and Span values), which can suggest our polymer-surfactant containing systems are mainly sterically stabilized. 

#### 3.1.3. Mucoadhesion Study

In order to improve the mucoadhesivity of the formulations, a mucoadhesive polymer, HPMC was added to the NLC systems. HPMC is a safe mucoadhesive polymer often used in ophthalmic formulations [6]. During our pre-formulation test, Methocel K4M polymer was chosen (Figure S1). The applied concentrations were 0.05 and 0.01, because higher polymer content resulted in the creaming of the nano-formulation. 

**Figure 2:**
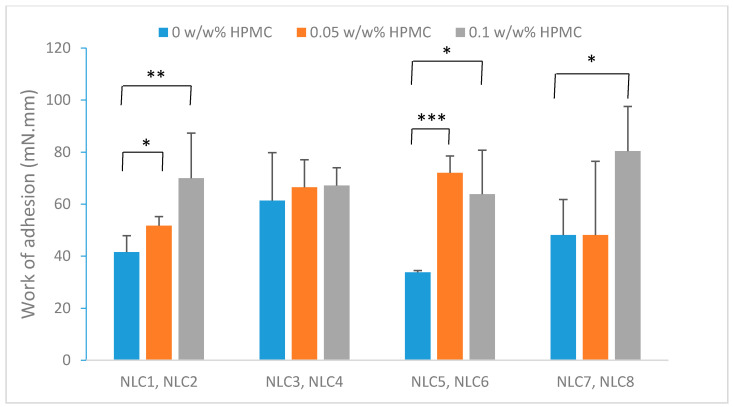
Comparison of mucoadhesion of the NLCs with and without polymer. (* *p* ≤ 0.05 significant; ** *p* ≤ 0.01 very significant; *** *p* ≤ 0.001 highly significant difference from NLC without polymer).

Because of its surface-active property [24], HPMC may accumulate at the lipid-water interface, resulting in a concentrated gel layer around the particles. This gel layer can to attach each particle separately to the mucosal surface. The mucoadhesion of the NLC compositions (Table 1) was compared with the same compositions without HPMC and then statistical analysis (T-test) was applied to evaluate the significance. It was found that there is a significant difference between samples with and without the mucoadhesive polymer (Figure 2) in the case of most compositions. Generally, the samples containing a higher surfactant amount (5%) (NLC3 and 4) displayed higher adhesive work values than the compositions with a lower surfactant amount, which can mean remarkable mucoadhesivity even without adhesive polymers. The possible cause of this phenomenon is the different interfacial layer. In these formulations, the addition of the polymer did not change the mucoadhesivity, which can be explained by the fact that the high surfactant concentration limits the orientation of the polymer to the surface of the NLCs. In our compositions, the polymer concentration did not change mucoadhesivity in the investigated polymer concentration range; there was no significant difference between the samples containing 0.05% and 0.10% polymer, which could be explained by the saturated interface of the NLCs, even at a low polymer concentration.

### 3.2. Cell Viability Assay

**Figure 3:**
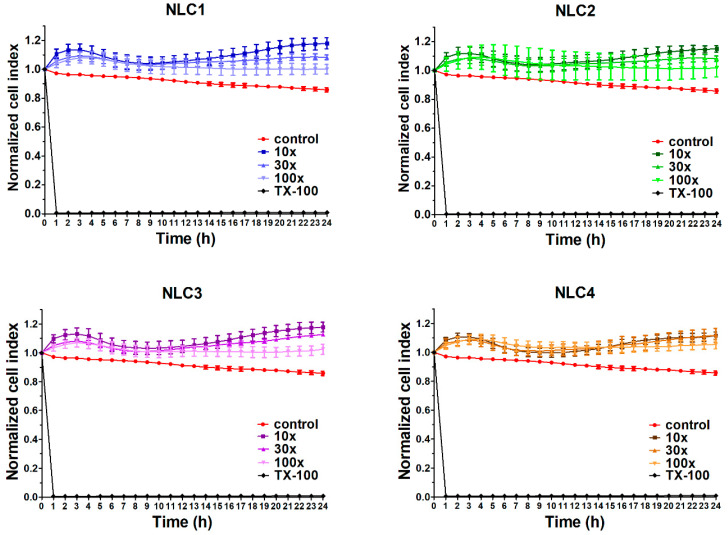
Cell viability of HCE-T cornea epithelial cells treated with different formulations measured by real-time electric sensing. Values are presented as means ± SD, *n* = 6–10. 10×: 10-times dilution; 30×: 30-times dilution; 100×: 100-times dilution; NLC, Nanostructured lipid carriers; TX-100, Triton X-100.

In order to investigate the potential toxicity of the formulations, cell viability assay was performed using impedance measurement. Real-time cell electronic sensing is a non-invasive, label-free, impedance-based technique to quantify the kinetics of proliferation, viability, and cellular reaction of adherent cells. This method can be successfully used to follow cell damage and/or protection in living barrier forming cells [22,25]. In our previous study [17], the non-toxicity of the applied emulsifier in the applied concentrations and the polymer free NLCs was already verified (using MTT assay), therefore in the present study, the full NLC systems (NLC1-4) and the polymer alone were evaluated. The impedance measurements did not show significant cell damage after treatment with different DXM containing NLC formulations (Figure 3). As a comparison, cells treated with the reference molecule Triton X-100 were lysed and impedance dropped to a minimum. DXM exerted a cell layer tightening effect that is visible in all NLC treatment groups as an increased level of impedance [26,27]. We found good correlation of cell impedance data with conventional cellular toxicity assays, like MTT and lactate dehydrogenase release tests and cellular morphology in a previous study [28].

**Figure 4:**
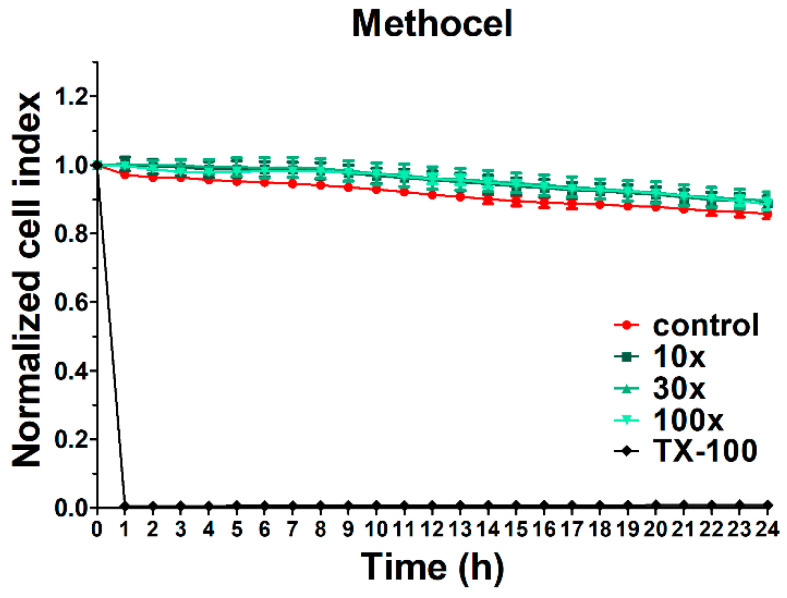
Cell viability of HCE-T cornea epithelial cells treated with Methocel measured by real-time electric sensing. Values are presented as means ± SD, *n* = 6–10. 10×: 10-times dilution; 30×: 30-times dilution; 100×: 100-times dilution; TX-100, Triton X-100.

Methocel did not significantly change the cell index of HCE-T cells, which indicates no cytotoxic effect on this cell type (Figure 4) similar to the tested NLCs. The reference compound, Triton X-100 detergent, caused cell death, as reflected by the decrease in impedance.

### 3.3. Immunohistochemistry

**Figure 5:**
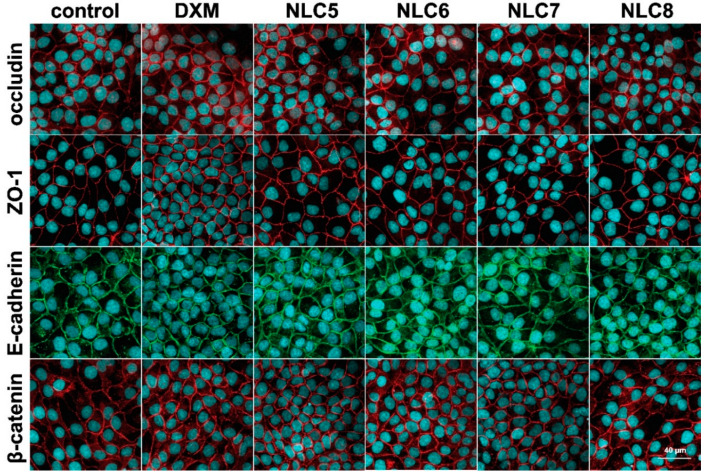
Effects of DXM alone and in formulations (10-times dilution) on junctional morphology of HCE-T corneal epithelial cells. Immunostaining for tight and adherens junction proteins occludin, zonula occludens-1 (ZO-1), E-cadherin and β-catenin after 1-h treatment. Red and green colors: immunostaining for junctional proteins. Blue color: staining of cell nuclei. Bar: 40 µm.

The cell viability test was supplemented with immunohistochemistry in order to clarify the effect of the formulations on the barrier function of human corneal epithelial cell layers. The HCE-T cornea epithelial cells formed a tight paracellular barrier visualized by the localization of the integral transmembrane tight junction protein occludin, the adherens junction protein E-cadherin and the cytoplasmic linker proteins associated to tight (ZO-1), and adherens junctions (β-catenin). No major morphological changes were observed in the treated groups. All junctional proteins were localized at the intercellular connections forming continuous pericellular belts in every group (Figure 5).

### 3.4. In Vitro Drug Diffusion Study

To investigate the release of DXM from our formulation, an in vitro dialysis bag method was used and the DXM concentration of each sample was determined by HPLC. The compositions were the same as in the factorial experimental design (NLC1-8). 

Considering the results of the factorial design, we can conclude that the emulsifier and the DXM concentration have a significant effect (significant factors are indicated in bold) on drug diffusion (diffused drug amount at 6 h, (C_DXM (6h)_)). The surfactant concentration has a significant negative effect on diffusion (Equation (12)), whereas the DXM concentration has a significant positive effect on diffusion.

C_DXM (6h)_ = 73.50 +1.64A − **14.40B** + **25.07C** − 1.24AB + 2.65AC − 3.11BC, (12)

**Figure 6:**
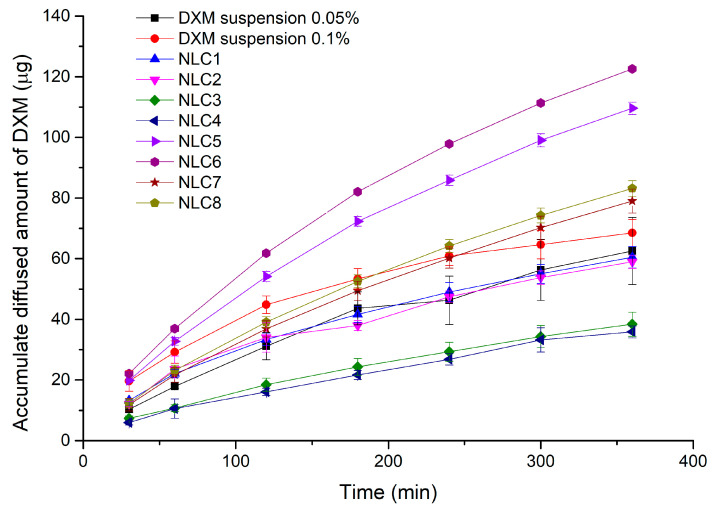
The diffused amount of DXM from NLC compositions through dialysis membrane.

The highest diffused amount of DXM was detectable in the case of NLC6 and 5 (Figure 6 and Figure S2), which contain a lower amount of surfactant, and as we demonstrated above, these formulations had the lowest entrapment efficacy (Table 2). This finding could show us that if less API is entrapped in the nanocarriers (more free DXM), a higher amount will diffuse through the synthetic membrane, and on the other hand, it can mean that the high emulsifier concentration (and/or high entrapment efficiency) in the system can have a restraining effect on DXM diffusion. At lower drug contents (NLC1-4 and 0.5% DXM suspension), the dilution of the acceptor phase during sampling can result in the total dissolution of DXM in suspension form, while the incorporation into a lipid carrier with high entrapment efficacy can hinder this dissolution process into the medium (as it was mentioned above). That is why we could observe a lower diffusion rate in our NLC formulations, which contain a high surfactant and a low API amount, compared with the conventional suspension form. This could explain why NLC3 and 4 with higher entrapment efficacy showed the lowest diffused amount of DXM (Table 2). 

### 3.5. Penetration Studies 

Permeability is the ability of drug molecules to penetrate trough the biological membranes. In our study, three different methods were applied and compared with each other, such as in vitro high throughput corneal-PAMPA; permeability study on cell culture; and ex vivo penetration study on porcine cornea followed by Raman correlation mapping. 

#### 3.5.1. Application of Corneal-PAMPA Model

**Figure 7:**
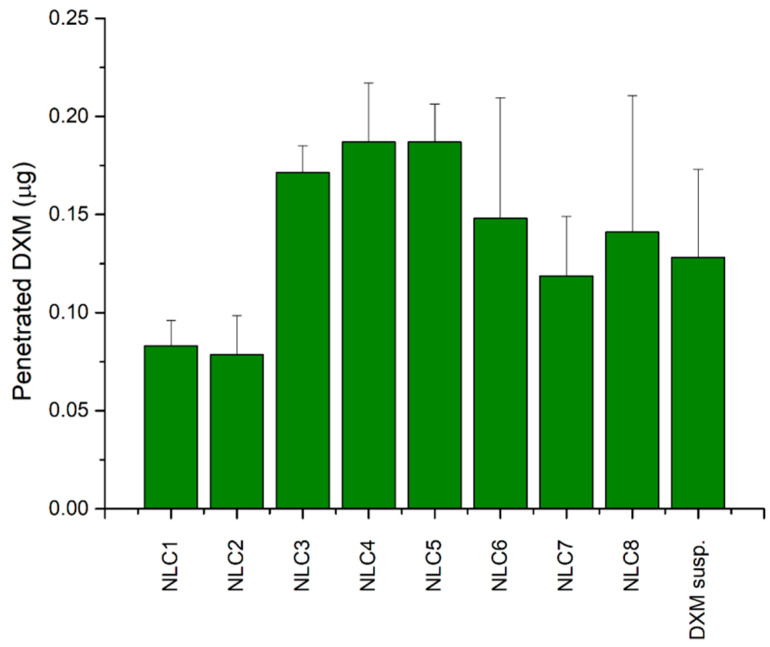
Comparison of the diffused amounts of DXM through the Corneal-PAMPA model.

In order to get more information about the expected amount of penetrated DXM through the cornea, a parallel artificial membrane permeability assay (PAMPA) was used. On the basis of the literature data, cornea-PAMPA can be successfully applied for the penetration study of ocular APIs as a fast, cost-effective screening method [20]. In our study, the penetration of DXM in different NLCs was evaluated and compared with DXM suspension (0.1%) in order to find correlation between the composition and the penetration ability of DXM. The highest penetrated DXM amount was detectable in the case of NLC3, 4, 5, and 6, and there was no significant difference between the penetrated amount of DXM in the case of DXM suspension and NLC1-8 (Figure 7). 

**Figure 8:**
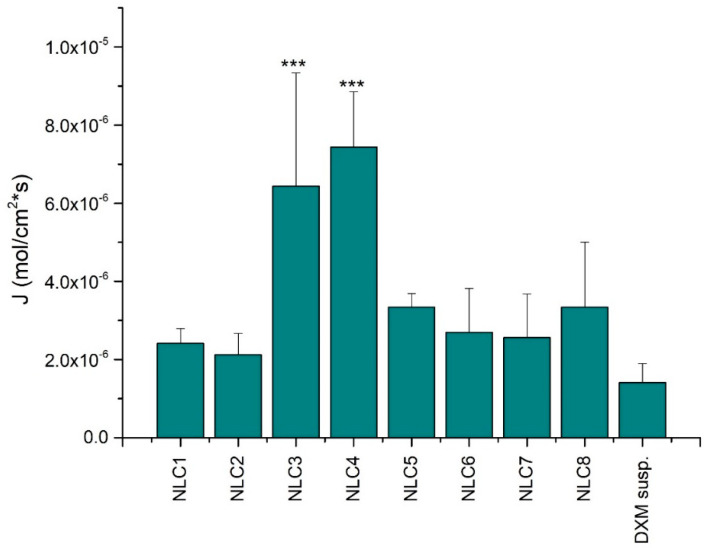
The flux of DXM from the NLC systems through the corneal-PAMPA model. Values are presented as means ± SD. Statistical analysis: ANOVA followed by Dunnett’s test. (*** *p* ≤ 0.001 highly significant difference from DXM suspension (DXM susp.)).

More relevant information can be concluded when the flux values are considered; in this case, the donor concentration of the applied formulations is considered (Figure 8). The comparison of the flux values of the NLC systems with the suspension indicated remarkable penetration when DXM was incorporated in nanocarriers. Surprisingly, NLC3 and 4 showed the highest penetration and a highly significant difference from the DXM suspension form. However, the NLC3 and 4 formulations showed a lower drug diffusion during the in vitro drug diffusion study. This contradiction may be explained by the interaction between the components of the NLCs and the cornea-PAMPA membrane. The applied emulsifier and lipids can modify the permeability of the impregnated PAMPA membrane, resulting in improved penetration. The largest alteration between the PAMPA and the in vitro drug diffusion study can be observed in the case of the compositions with higher surfactant concentration, which can suggest that the interaction occurs between the emulsifier and the membrane, resulting in improved PAMPA penetration. 

#### 3.5.2. Permeability Study on Cell Culture Model

**Figure 9:**
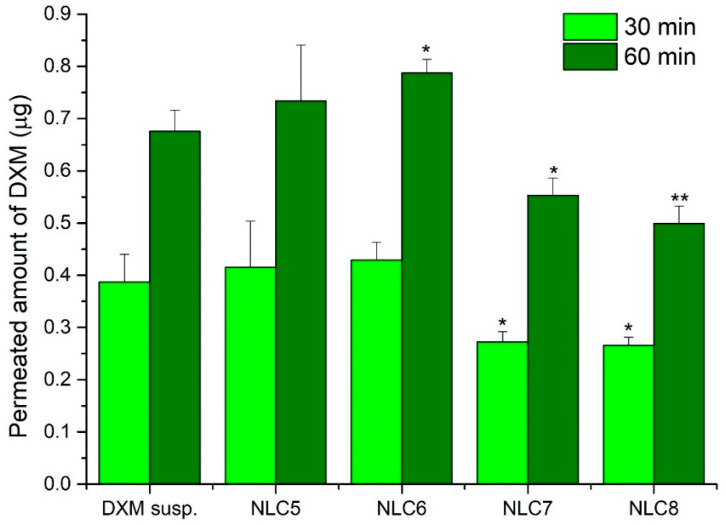
The penetrated amount of DXM through HCE-T cells after 30 min and 60 min. Values are presented as means ± SD. Statistical analysis: ANOVA followed by Dunnett’s test. (* *p* ≤ 0.05 significant; ** *p* ≤ 0.01 very significant difference from DXM suspension (DXM susp.)).

The permeability of DXM from suspension and different NLC formulations was tested on the HCE-T cell model as well. It can be seen that the highest amount diffused in the case of the NLC5 and NLC6 formulations, while the NLC7,8 formulations showed a lower amount of diffused DXM (Figure 9). After 60 min, the diffused amount of DXM was significantly higher for NLC6 than for the suspension form. When there was higher free concentration in the donor phase, the amount of diffused DXM was also higher. This tendency is similar to the one seen during the previous investigation with dialysis membrane. This may suggest that if the entrapment efficiency is higher, DXM can diffuse in a lower amount from the NLC systems.

**Figure 10:**
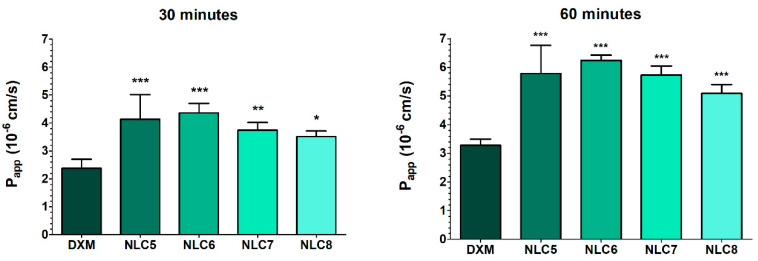
Apparent permeability coefficients (P_app_) for DXM suspension (DXM) or in different formulations (10-times dilution of NLCs) measured across HCE-T corneal epithelial cell layers after 30 and 60 min of incubation. Values are presented as means ± SD, *n* = 4/group. Statistical analysis: ANOVA followed by Dunnett’s test. (* *p* ≤ 0.05 significant; ** *p* ≤ 0.01 very significant; and *** *p* ≤ 0.001 highly significant difference from DXM suspension (DXM susp.)).

Considering the permeability (P_app_) values after treatment, all NLC formulations showed higher values compared to the suspension (30 min: 2.4 ± 0.3 × 10^−6^ cm/s; 60 min: 3.3 ± 0.2 × 10^−6^ cm/s), which indicates a better penetration of the API using nano lipid compositions (Figure 10). 

**Figure 11:**
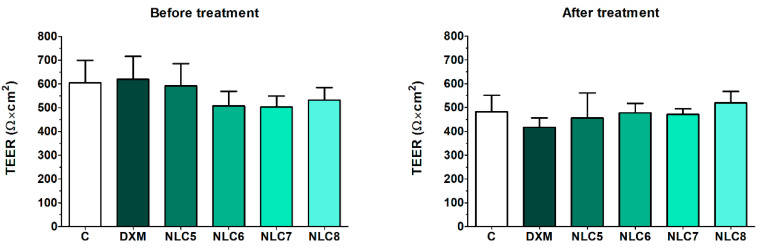
Transepithelial electrical resistance (TEER) values of HCE-T cell layers before and after the permeability experiment. Values are presented as means ± SD, *n* = 4/group. Statistical analysis: 2-way ANOVA followed by the Bonferroni test.

The barrier properties were investigated after the permeability study measuring the TEER values. HCE-T cell layers showed high TEER values, indicating tight barrier properties (Figure 11) and suitability for the permeability assay. After 60 min of treatment, the TEER values did not decrease significantly, and the different formulations did not change the TEER values significantly compared to the relevant control groups, suggesting no damaging effects of the formulations on the epithelial cell layers. 

**Figure 12:**
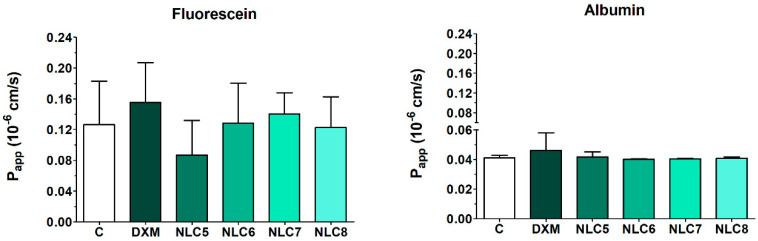
Apparent permeability coefficients (P_app_) of passive paracellular permeability markers fluorescein and Evans blue labeled albumin measured on HCE-T corneal epithelial cell layers. Values are presented as means ± SD, *n* = 4. Statistical analysis: ANOVA followed by Dunnett’s test.

These good barrier properties were confirmed with the permeability measurements of different indicator molecules (fluorescein and albumin). The human corneal epithelial culture model showed low permeability values for fluorescein (0.126 ± 0.057 × 10^−6^ cm/s) and for albumin (0.041 ± 0.002 × 10^−6^ cm/s) hydrophilic paracellular marker molecules, indicating a tight barrier both in the control group and in cell layers after the DXM penetration experiment (Figure 12). These values show that the epithelial barrier was not affected by the formulations, as it was also presented in the immunohistochemistry assay earlier. The P_app_ for lipophilic DXM (Figure 3) was 58 times higher than the P_app_ for the large marker albumin, and 19 times higher than the P_app_ for the small hydrophilic marker fluorescein.

One of the most interesting points in the present study was that the DXM containing NLCs did not affect intercellular junctions in our culture model of corneal epithelial cells. Based on the literature, both clathrin and caveolin mediated endocytosis pathways are involved in the uptake of solid lipid nanoparticles in cultured retinal pigment epithelial cells [29] as well as in a rat intestinal ex vivo model [30].

#### 3.5.3. Correlation of the Different Penetration Models

In order to evaluate the penetration studies, the correlation of the penetrated amount of drug through different membranes was analyzed. Correlated results may confirm the relevance and applicability of the models and can help to choose the most suitable one to predict the bioavailability of the API in NLCs.

**Figure 13:**
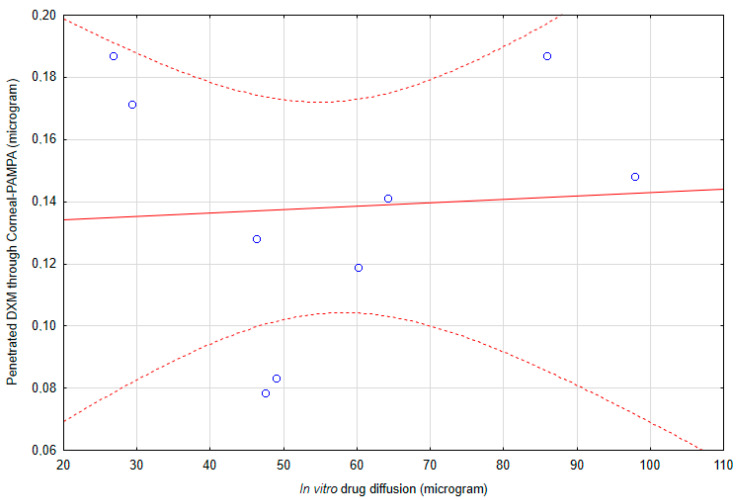
The correlation of the in vitro diffusion study and penetrated amount of DXM through Corneal-PAMPA.

The results of the in vitro diffusion study and penetrated amount of DXM though Corneal-PAMPA do not correlate with each other (Figure 13). The correlation coefficient was 0.06522, which means that there is no correlation between the results of penetration and diffusion studies.

**Figure 14:**
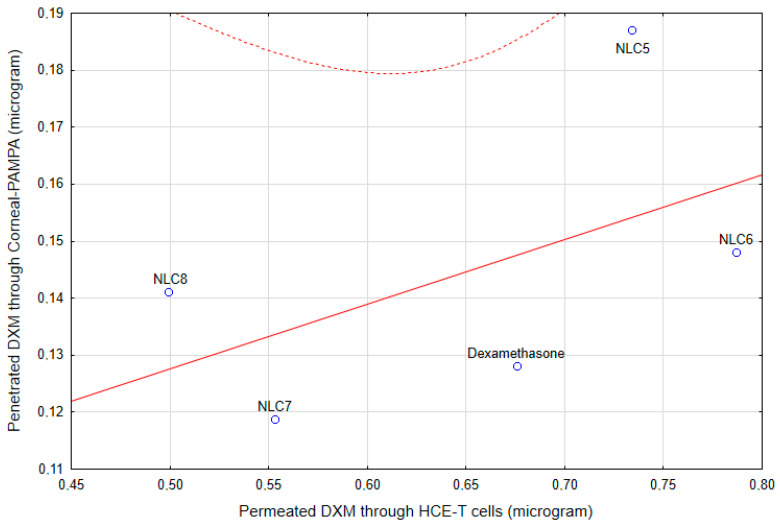
The correlation of the HCE-T cell line permeability and the Corneal-PAMPA model.

The correlation coefficient between the results of the penetrated DXM though HCE-T cells and the penetrated amount though Corneal-PAMPA is 0.52248, which indicates a moderate correlation [31] between these two models (Figure 14). However, as was mentioned above, the component of the NLC can modify the PAMPA membrane, which can alter the permeability in this model, while the cell membrane remained the same in the case of the cell penetration study, which can explain this moderate correlation.

**Figure 15:**
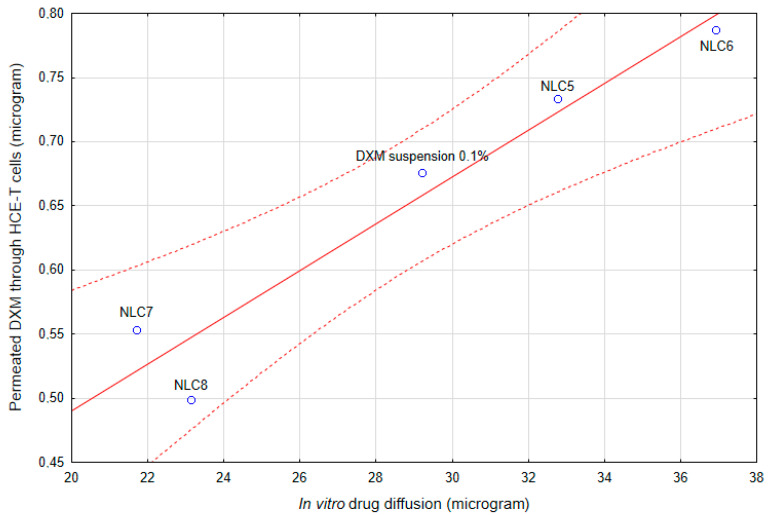
The correlation of the in vitro diffusion study and HCE-T cell line permeability.

Surprisingly, the correlation between the in vitro diffusion study and the HCE-T cell line permeability study was very high (Figure 15). The correlation coefficient was 0.96595, which indicates a strong correlation. As was presented, the formulations did not change the barrier function of the corneal epithelial cells (immunohistochemistry, P_app_ of fluorescein and albumin); the main rate-limiting factor for the penetration of DXM in NLC formulations is the drug release from the nanocarriers. 

The correlation study indicated altered results for the PAMPA model, namely the applied surfactant and lipid can modify the PAMPA membrane, which can result in inappropriate conclusions. This observation can highlight the possible limited applicability of the corneal-PAMPA model in the case of lipid compositions. 

As a practical conclusion, in the future, the in vitro drug release study can offer a possibility to compare the bioavailability of further modifications of this type of NLCs.

#### 3.5.4. Ex Vivo Raman Correlation Mapping

Based on the drug diffusion study and the cell line penetration test, NLC6 was selected for the semi-quantitative Raman mapping examination. The chosen formulation NLC6 was compared with its polymer free version. The porcine corneas were treated with the formulations with NLC6, and without polymer; and the depth of penetration was analyzed. 

**Figure 16:**
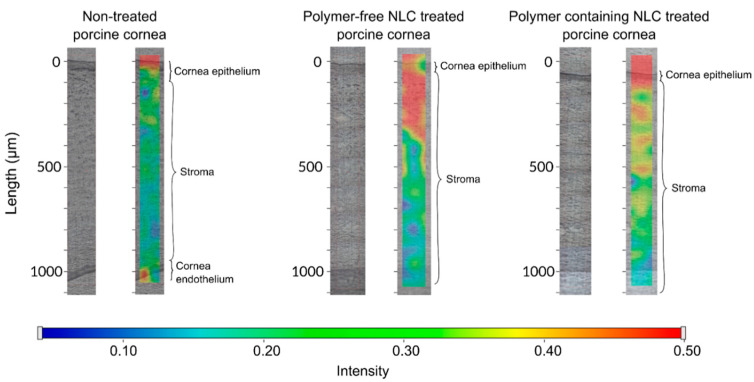
The Raman correlation map of NLC6 and the polymer-free version of the composition.

The NLC6 and the same formulation without HPMC-treated porcine cornea specimen was compared with the non-treated cornea specimen. The spectrum of NLC6 was used for the cornea distribution correlation maps, except in the case of the polymer-free formulation treated cornea specimen, when its spectrum was applied for creating the correlation map (Figure 16). In the case of the polymer-free NLC, we can observe a significant amount of components (surfactant, lipids) of NLCs in the stroma layer (300 µm penetration depth) of the cornea. 

As for the polymer containing NLC (NLC6), a very remarkable Raman intensity can be seen on the surface of the cornea and in the upper part of the stroma, but the latter is weaker than in the case of the NLC without polymer. Thanks to its adhesive properties, NLC6 might form a very significant depot on the corneal surface, and it presents fewer transitions of components through the epithelial layer into the hydrophilic stroma. The accumulation of the hydrophilic polymer at the nano lipid carrier surface can change the polarity of the lipid surface and increase particle size; therefore, these modifications can lead to lower but still remarkable penetration of some NLC components across the lipophilic corneal epithelial cells. 

## 4. Conclusions

In the present study, DXM-loaded NLCs were successfully combined with a mucoadhesive polymer. In our formulations, a mucoadhesive gel layer might be formed at the interface of the nanoparticle, thanks to the amphiphilic characteristics of HPMC.

Based on the results of the mucoadhesive studies, the incorporation of HPMC increased the mucoadhesive properties compared with the polymer-free formulations, even at very low polymer concentration.

The characterization of nanoparticles (Z_ave_, PDI, d(0.5) and Span values) indicated that the preparation with the lowest absolute ZP values had the highest stability, which may suggest that our polymer-surfactant containing systems are mainly sterically stabilized. 

The biocompatibility of the NLC systems was evaluated using toxicity tests on HCE-T cells with impedance measurement and immunoassay analyses. These measurements indicated no cell damage after treatment and the compositions did not have any harmful effects on cell junctions. 

The drug release study of DXM showed increased drug diffusion from the NLC systems compared with conventional suspension forms, and it could also be concluded that the formulation with very high entrapment efficiency can hinder drug release from the nanoparticles. These results were in correlation with the cell permeability assays, where the optimized NLCs presented significantly better permeability across HCE-T cells. 

The mucoadhesivity and penetration enhancing effects of our NLCs were presented in an ex vivo penetration-retention study through porcine cornea with Raman correlation mapping. The non-treated and treated (NLC with and without polymer) porcine corneas were compared. The results showed that the NLC systems with HPMC polymers could create a depot on the surface of the cornea. This might suggest a potential sustained release and increased residence time of DXM using this type of polymer-modified NLC systems.

## Figures and Tables

**Table 1:** The composition of the nanostructured lipid carriers (NLCs).

	Polymer Concentration (*w*/*w*%)	Surfactant Concentration (*w*/*w*%)	DXM Concentration (*w*/*w*%)
NLC1	0.05	2.5	0.05
NLC2	0.10	2.5	0.05
NLC3	0.05	5.0	0.05
NLC4	0.10	5.0	0.05
NLC5	0.05	2.5	0.10
NLC6	0.10	2.5	0.10
NLC7	0.05	5.0	0.10
NLC8	0.10	5.0	0.10

**Table 2:** The particle size, zeta potential, PDI and entrapment efficacy of the NLC compositions (mean and ± SD).

	After 1 Day				After 1 Week		
	Z_ave_ (d.nm)	PDI	ZP (mV)	EE%	Z_ave_ (d.nm)	PDI	ZP (mV)
NLC1	202.33 ± 0.70	0.42 ± 0.008	−10.0 ± 0.3	83.50	285.67 ± 4.06	0.55 ± 0.01	−10.0 ± 0.3
NLC2	192.17 ± 2.86	0.30 ± 0.040	−11.9 ± 0.4	83.47	241.13 ± 1.80	0.46 ± 0.02	−9.2 ± 0.2
NLC3	128.87 ± 3.96	0.35 ± 0.015	−3.7 ± 0.2	95.07	141.07 ± 1.86	0.46 ± 0.02	−13.3 ± 0.3
NLC4	96.46 ± 0.63	0.20 ± 0.006	−3.3 ± 0.4	91.06	122.50 ± 0.50	0.43 ± 0.01	−4.9 ± 0.6
NLC5	188.27 ± 2.70	0.29 ± 0.006	−10.2 ± 0.1	86.19	264.77 ± 7.21	0.49 ± 0.02	−9.4 ± 0.2
NLC6	200.70 ± 7.63	0.34 ± 0.039	−7.4 ± 0.1	86.94	270.07 ± 6.66	0.49 ± 0.03	−15.8 ± 0.6
NLC7	113.13 ± 0.81	0.25 ± 0.005	−8.6 ± 0.2	91.78	143.63 ± 3.97	0.44 ± 0.04	−9.5 ± 0.6
NLC8	103.83 ± 0.46	0.23 ± 0.006	−6.2 ± 0.2	91.02	137.83 ± 0.38	0.45 ± 0.01	−7.7 ± 0.2

**Table 3:** The results of laser diffraction-based particle size analysis.

	After 1 Day				After 1 Week			
	d(0.1)	d(0.5)	d(0.9)	Span Value	d(0.1)	d(0.5)	d(0.9)	Span Value
NLC1	0.075	0.125	0.245	1.355	0.107	1.523	44.681	29.269
NLC2	0.071	0.122	0.229	1.293	0.091	0.303	35.697	117.694
NLC3	0.074	0.113	0.190	1.026	0.066	0.135	1.123	7.819
NLC4	0.075	0.111	0.168	0.831	0.063	0.122	0.244	1.486
NLC5	0.074	0.123	0.227	1.241	0.097	0.415	39.093	93.901
NLC6	0.070	0.122	0.248	1.461	0.097	0.393	36.777	93.387
NLC7	0.079	0.123	0.266	1.532	0.087	0.266	13.672	51.152
NLC8	0.074	0.118	0.212	1.169	0.065	0.130	0.525	3.521

## Supplementary Materials

The following are available online at https://www.mdpi.com/1999-4923/12/7/682/s1, Figure S1: The adhesive work of different Methocel polymers, Figure S2. The accumulate diffused rate of DXM from NLC compositions through dialysis membrane.
