# Supplementary material for: Development and Characterization of Potential Ocular Mucoadhesive Nano Lipid Carriers Using Full Factorial Design
Source: Pharmaceutics. 2020 Jul 20;12(7):682. doi: 10.3390/pharmaceutics12070682 (PMC7408368; doi:10.3390/pharmaceutics12070682)
Supplement: Supplementary file 1 [file pharmaceutics-12-00682-s001.pdf]

pharmaceutics

Supplementary Materials: Development and Characterization of Potential Ocular Mucoadhesive Nano Lipid Carriers Using Full Factorial Design

Eszter L. Kiss, Szilvia Berkó, Attila Gácsi, Anita Kovács, Gábor Katona, Judit Soós, Erzsébet Csányi, Ilona Gróf, András Harazin, Mária A. Deli, György T. Balogh and Mária Budai-Szűcs

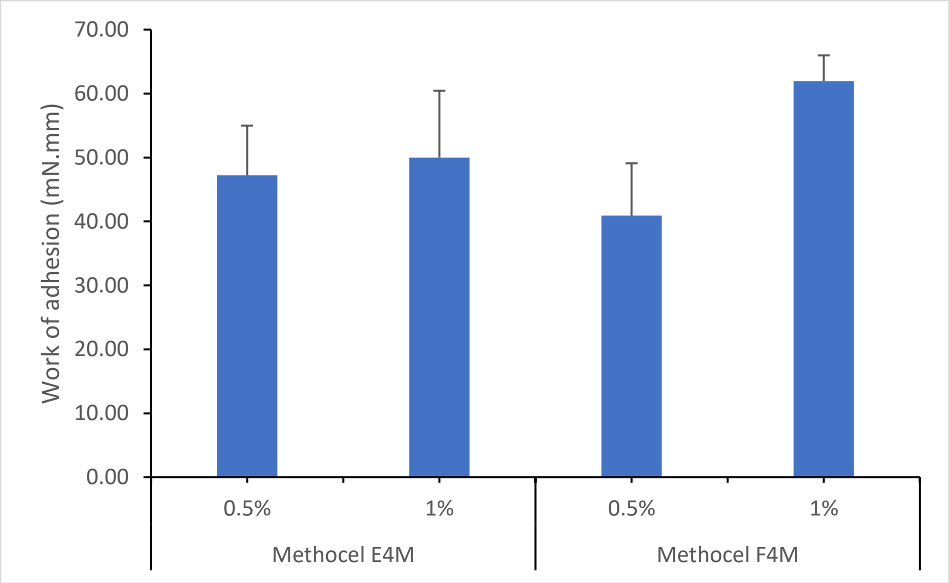

Figure S1. The adhesive work of different Methocel polymers.

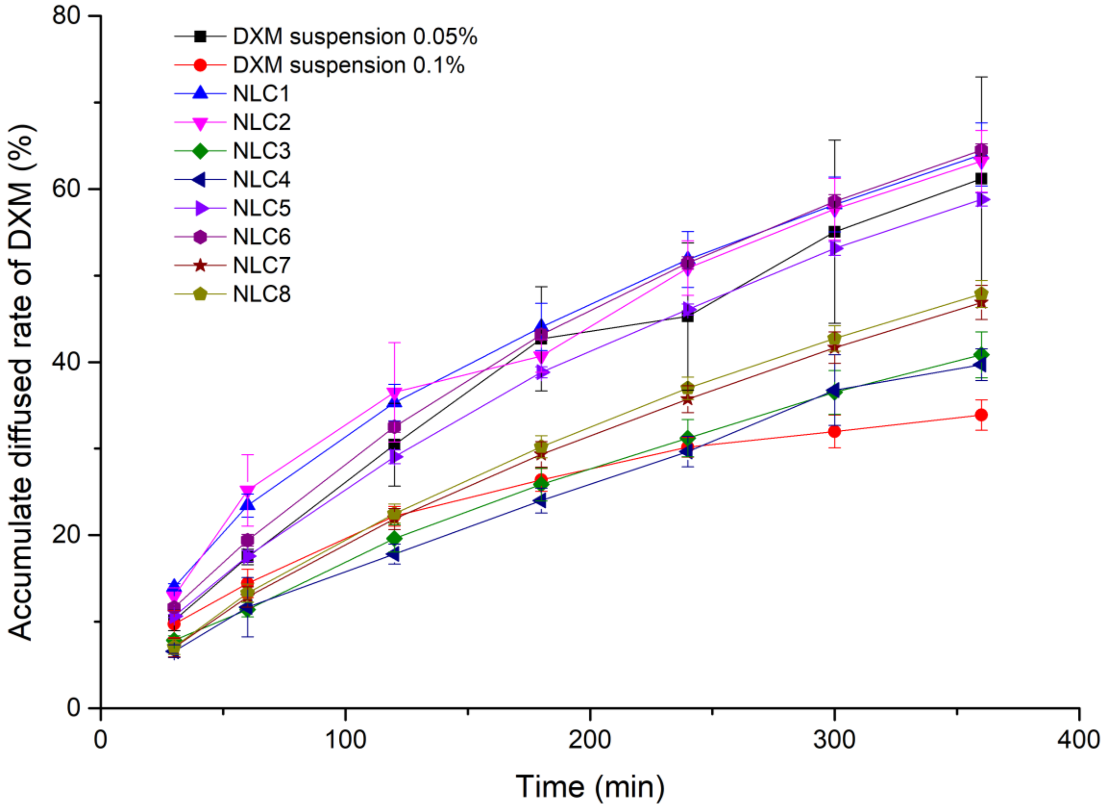

Figure S2. The accumulate diffused rate of DXM from NLC compositions through dialysis membrane.
